# Supplementary material for: Are carbon emissions trading and green financial instruments synergistic? -Comprehensive quantitative research based on content analysis
Source: PLoS One. 2024 Mar 7;19(3):e0298601. doi: 10.1371/journal.pone.0298601 (PMC10919601; doi:10.1371/journal.pone.0298601)
Supplement: S1 Data — (ZIP) [file pone.0298601.s001.zip › Data/地级市Z_F工作报告.zip!/║■▒▒/╦μ╓▌╩╨/└┤╫╘╣1⁄2╓┌║┼í╛┬φ┐╦╩2╛▌═°í┐_2009.docx]

各位代表：

现在，我代表市人民政府向大会作政府工作报告，请予审议，并请市政协委员和各位列席人员提出意见。

一、2008年工作回顾

2008年，是极不平凡的一年，是全市人民经受严峻考验并取得丰硕成果的一年。一年来，我们在省委省政府和市委的正确领导下，坚持以科学发展观为统领，以新一轮思想大解放为动力，全力应对自然灾害，积极顺应宏观调控，着力化解国际金融危机等不利因素影响，克难攻坚，顽强拼搏，开拓进取，奋力赶超，主要经济指标高于去年同期增幅、高于全省平均水平，圆满完成了市二届人大二次会议确定的各项任务，经济社会实现了又好又快发展。

——国民经济快速增长。预计全市地区生产总值可突破300亿元，同比增长14.5%。农业农村经济稳步发展。实现农业增加值68亿元，同比增长5.5%。农业生产喜获丰收，主要农产品普遍增产，食用菌、马铃薯、油桃等特色板块继续扩大。畜牧产业强势推进，建成标准化养殖小区83个。农业产业化水平进一步提高，同星农业被认定为国家级龙头企业；三友食品出口创汇过5000万美元，成为全省第一大农产品出口企业。新造林和因灾受损补植面积18万亩，集体林权制度改革扎实推进，已确权面积达到80%，森林资源管护得到加强。完成各类水利工程1.5万处，解决了13.1万人的饮水安全问题，大中型水库除险加固、大型灌区续建配套和节水改造工程按计划推进，水库管理体制改革基本完成。投资近4亿元，修建公路811公里，其中通村水泥路582公里，行政村客车通达率达到86%。工业经济再创新高。预计实现规模工业增加值85亿元，同比增长22%。增长结构更趋优化，五大支柱产业强劲支撑，增加值占规模工业总量的71%。新建投资过千万元的工业项目94个，规模企业净增58家，亿元企业净增12家，提前3年完成市二次党代会提出的“规模企业突破500家、亿元企业突破50家”的目标。工业企业改制处于收尾阶段，国资监管得到加强。第三产业持续发展。积极创建“中国优秀旅游城市”，我市被纳入鄂西生态文化旅游圈发展战略，大洪山、神农故里核心景区、旅游专线等重点景区和旅游配套项目建设实现重大突破。成功举办首届编钟文化节暨编钟出土30周年系列纪念活动，成功举办桃花节、兰花节、银杏节、香菇节。旅游产业持续升温，全年接待游客突破400万人（次），旅游综合收入20亿元。以“万村千乡市场工程”为载体，积极推进城乡市场对接，建成“农家店”183家。加强市场运行监测，启动价格干预机制，保证了雪灾等特殊时期的市场供应和物价稳定。深入开展农资、卷烟、奶制品、生猪屠宰、医药等专业市场整顿，市场秩序进一步规范。建筑和房地产业有序发展，典当、信息、物流配送、电子商务等新兴服务业和现代物流业蓬勃发展。

——三驾马车强劲驱动。投资、消费、出口三大需求增长较快。累计完成全社会固定资产投资132.5亿元，同比增长30%，其中城镇以上固定资产投资110亿元，增长40%。投资结构明显改善，工业项目投资占城镇以上投资的比重进一步提高，入库国家和省投资项目181个。招商引资取得突破，全市共引进内联资金40亿元，同比增长28%；实际利用外资3900多万美元，增长22%。消费品市场繁荣活跃，预计实现社会消费品零售总额150亿元，同比增长23%。对外贸易亮点纷呈，呈现农产品、机电、纺织、医药化工产品竞相出口的多元化格局，预计实现出口3.22亿美元，同比增长60%，其中香菇出口首次超过1亿美元，机电产品出口同比增长4.8倍。

——财政实力逐步提升。狠抓财源建设，加强税收征管，财政形势好于预期，完成财政总收入14.7亿元，同比增长18.8%，其中一般预算收入6.68亿元，增长22%。完善市经济开发区、曾都经济开发区财政体制，提高中小企业担保公司融资规模，财政保发展、保民生、保稳定的能力得到提升。金融保险业健康运行，12月末金融机构各项存款余额可达274.8亿元，比年初增加48.7亿元，增长21.5%；贷款余额88.9亿元，比年初增加13.1亿元，增长17%。

——城乡面貌显著变化。城市总体规划修编已报经省专家委员会评审通过，明确了“东拓、西抑、南优、北调”和“一河两岸，轴线组团”的城市发展布局。加大违规违法建筑拆除力度，城市规划区建筑秩序稳定好转。严格控制土地一级市场，严格规范土地管理程序，确保一个渠道进、一个漏斗出和有偿使用、公平有序。依法经营城市无形资产，全面推行了出租车经营权出让。坚持量力而行、尽力而为，年初确定的十大重点工程除体育馆因资金制约未能“基本完成”外，其余9件全部完成，白云湖水库开始蓄水，绕城公路建成通车，污水处理厂投入运行，擂鼓墩大道、解放路中段、白云湖东大堤、文峰路等一批城区道路改造全面竣工。新增城市绿地面积58万平方米，新建了一批路灯、公厕、垃圾中转站等市政设施。坚持精细化管理的原则，纵深推进“三城同创”，被正式授予省级文明城市、省级卫生城市，“楚天杯”创建工作受到省考核组肯定。优先发展城市公交，“禁摩”扩面顺利推进。大力开展村庄整治，全面实施“清洁工程”，140个建制村基本完成整治任务，重点镇和示范村建设力度加大，2个乡镇被授予国家级文明乡镇，10个村镇被授予省级文明村镇。

——社会建设加快推进。以和谐随州、平安随州建设为主题，协调发展各项社会事业，全力维护社会稳定。加快提高自主创新能力，争取省级以上科技项目21个，申请专利190项，新增高新技术企业4家，建立特色农业科技示范基地21个。义务教育“两免一补”政策全面落实，办学条件不断改善，教育质量继续提高，高考万人上线率位居全省前列，职业教育蓬勃发展。提高公共卫生突发事件应急能力，重大传染病防治经验在全国推广，新型农村合作医疗参合率85%以上，社区医疗卫生救治能力不断增强。进一步提升公共文化服务水平，以迎奥运为主题的群众性文化体育活动广泛开展，博物馆、白云书院顺利建成并免费对市民开放；完成213个自然村广播电视“村村通”建设，新增有线电视用户2.8万户，改造扩建乡村文化站10个。城镇新增就业2万人，帮扶下岗失业人员再就业5000多人，农村劳动力培训转移就业3.2万人，全市社保扩面新增4万人，城镇居民基本医疗保险、被征地农民养老保险、农民工医疗和工伤保险都取得突破性进展。狠抓节能减排，关停污染企业13家，单位生产总值能耗、二氧化硫和化学需氧量排放总量三项约束性指标全面完成。人口和计生工作继续保持低生育水平，当年出生人口性别比达到省定目标。着力在耕地保护、节约集约利用土地和矿产资源开发整合上下功夫，国土资源发挥了保障发展的重要作用。以安全生产“隐患治理年”为主线，完善工作机制，加强排查整改，安全生产形势稳定好转。以保障人民群众生命财产安全为目的，健全社会治安防控体系和公共安全应急处理机制，扎实推进平安随州建设，确保了北京奥运会期间秩序良好，有效维护了全市社会稳定。妇女、儿童、老龄和残疾人事业较快发展。

——人民生活继续改善。预计实现农民人均纯收入4967元，同比增加790元；城市居民人均可支配收入11630元，同比增加1605元。切实解决城市低收入群体和农村受灾群众住房困难，加快经济适用房、廉租房建设，600套廉租房即将交付使用，全市所有雪灾倒房户都实现了住有新居，25个贫困村“整村推进”扶贫工作顺利完成。一次性拨付城市低保居民年度用电优惠资金130万元。设立市慈善医院，建成市首家孤残儿童福利院。强化执政为民宗旨，努力为民多办实事，2008年《政府工作报告》中承诺的10件实事顺利实施、全面完成。

——精神文明建设、民主法制建设和政府自身建设进一步加强。深入开展文明城市、文明村镇、文明行业、思想道德建设等群众性精神文明创建活动，公民思想道德素质得到提升，涌现出一批精神文明建设的先进典型。积极投入抗震救灾，社会各界向四川灾区捐款捐物价值2500多万元，我市被评为全省抗震救灾工作先进集体；灾后援建工作进展顺利。坚持依法治市，严格依法行政，加强文明执法教育，各级政府和政府部门自觉接受人大法律监督、政协民主监督、新闻舆论监督以及社会公众监督，扎实办好人大建议和政协提案。强化行政执法监督、审计监督、行政监察和案件查处工作，政府系统党风廉政建设进一步加强。不断提高政府执行力和行政效能，电子政务在政府系统开始试运行。全面完成第七届村委会换届选举工作，积极推行“逢四说事、有话就说”制度，基层民主进一步扩大。高度重视信访工作，最大限度地化解不和谐、不稳定因素。加强了与各民主党派、工商联和工会、共青团、妇联等人民团体的联系。努力做好国防教育、人民防空、民兵预备役和双拥工作，人事、编制、司法、粮食、物价、工商、统计、国调、对台、气象、档案、地方志以及民族宗教、外事侨务、食品药品监督、质量技术监督、信息产业等各项工作都取得了新的成绩。

各位代表，回顾过去一年的工作，我们深感：发展令人鼓舞，成绩来之不易。这是省委省政府和市委正确领导的结果，是市人大、市政协监督支持的结果，是全市人民团结奋斗的结果。在此，我代表市人民政府，向全市人民、各级干部，向驻随部队、武警官兵和政法干警，致以崇高的敬意！向各人民团体、民主党派、工商联和驻随单位，向对政府工作给予大力支持的人大代表、政协委员和社会各界人士，向所有关心、支持、帮助随州发展的海内外朋友，表示衷心的感谢！

在肯定成绩的同时，我们也清醒地看到，我市经济社会发展中长期积累的一些深层次矛盾和问题还未得到根本解决，主要表现在：思想解放不够，内陆意识还一定程度存在，尚未形成致力发展、全民创业的浓厚氛围；引进的大财团、大项目少，招商引资的力度有待进一步加强；国民经济结构不优，三次产业占比不合理，工业多点支撑的格局尚未形成、支柱地位有待进一步提升；与同类城市的差距不仅没有缩小，反而有拉大的趋势，赶超发展的压力进一步加大；少数部门和单位抓落实不够，主动服务意识不强，经济发展环境有待进一步优化。对此，我们一定高度重视，在今后工作中认真加以解决。

二、2009年的形势和任务

2009年是新中国成立60周年，是全面贯彻落实党的十七大、十七届三中全会精神的重要一年，也是国家扩大内需政策集中实施和我们应对国际金融危机、努力保持经济平稳较快增长的关键一年。

在国际金融危机继续扩散和蔓延、外部环境趋紧的影响下，我国经济下行的风险比预想的更加严重，经济下滑已经成为经济运行的主要矛盾，今年可能是进入新世纪以来我国经济发展最困难的一年。在全国全省经济放缓的大背景下，各级政府和全体政府工作人员，必将高度重视金融危机给我市经济社会发展带来的不利影响，准确地把握宏观形势，清醒地认识我们所面临的危机与挑战：一是我市经济总量小，抗风险能力较弱。由于我市经济规模小，一个较小的“增量”，一旦遇到调整冲击，就可能对经济“存量”的结构和效益产生重创。二是经济下行开始显现，保增长异常艰巨。从去年11月份开始，我市经济增速开始减缓，元至12月平均规模工业增加值较元至10月回落了2.3个百分点，地方一般预算收入回落2.8个百分点；尤其是部分重点企业出现经营困难，25家重点企业增加值同比仅增长5.3%，回落4.8个百分点。物价出现新变化，生产经营领域的利润空间大幅缩小，倒挂现象进一步扩大。三是外贸出口受到冲击，外向型企业面临前所未有的压力。纺织、农业、机电等产品国际市场空间受限，绝大多数企业开工不足，有的已经处于停产半停产和亏损状态。四是外出务工人员大量回流，就业再就业形势十分严峻。截至去年底，全市已经回流农民工6万多人，占外出务工人员总数的15%，而市内企业用工需求有限，随着农民工回流的大规模发生，将会给就业再就业工作带来很大影响，并可能由此产生一系列社会问题。五是社会消费预期下降，提振市场信心任务十分繁重。金融危机弱化了城乡居民消费预期，加深了对实体经济的影响，2008年11月居民消费价格指数比上年同期上涨2.1%，这是自2007年6月以来连续18个月最低的，月涨幅首次跌至3%以下。居民消费意愿下降，内需不振，导致相关行业发展受到严重影响。当前我们面临着尤为严峻的发展形势，今年可能是随州建市以来最艰难的一年。

“百年一遇”的金融危机，是对企业抵御风险能力的检验，是对各级政府执政能力的检验，更是对我们贯彻落实科学发展观、加快“四个随州”建设实践能力的检验。危难时刻，紧要关头，要提振经济、度过危机，关键是要准确研判形势，重建发展信心，“信心比黄金、货币更重要”。客观、全面地分析环境和形势，当前我市的发展虽然面临很大的压力，但也蕴藏着极大的潜力，我们完全具备危中求机、逆势而上的优势和条件：一是经济发展的基本面依然向好。从基础条件看：随州紧临武汉城市圈，交通发达，拥有两张世界级名片，历史文化底蕴深厚，生态环境较好，生产要素成本也相对较低。从经济指标看，去年我市拉动经济增长的 “三驾马车”一齐发力，主要经济指标呈现出“两个高于”的特点，全市经济保持了稳健发展态势。从发展势头看：经过近年来不断调整优化经济结构，我市五大工业支柱产业支撑作用明显增强，以特色农业为主导的农村经济增长格局已经形成，县域经济迅猛发展、势头强劲。二是国家启动内需的政策对我市相对有利。国家实施积极的财政政策和适度宽松的货币政策，两年4万亿的投资拉动，以及去年四季度1000亿项目资金的分解落实，必将在全国各地掀起新一轮抢抓政策机遇，加快项目建设的高潮，专用汽车产业具有寻求差异化发展的市场空间；随州作为农业大市，可以承接更多的惠农支持，可以争取更多的惠农补贴；丰富的旅游资源，有利于抢抓省政府突破性发展旅游业的扶持政策，把随州建设成为 “两圈”融合的最佳结合部和鄂西生态文化旅游圈的重要门户，促进产业结构的进一步调整。三是我市区位条件和优势产品具有抗风险性。金融危机的波及和蔓延有一定的规律，越是发达地区、开放地区和经济外向度高的地区，冲击越深、影响越大。我市地处内陆，经济外向度、金融依存度较低，受到的冲击相对较小。同时，我市的产品结构也具有独特的属性，同是汽车产业，专用汽车比乘用车受到的冲击相对要小；从出口结构看，我市农产品出口主要集中在亚太市场，机电产品出口主要集中在中东和非洲市场，欧美市场只占我市贸易总量的20%左右，目前受金融海啸的影响尽管存在，但相对有限。四是 “草根经济”的支撑作用明显。我市有一支经过市场竞争锤炼的企业家队伍，长期的市场积累，使他们具备了较为成熟的经营管理理念和市场驾驭能力；经过市场经济的磨炼之后，生存和成长起来的本土企业，大多具有较强的韧性，具备应对困难、抗击风浪的能力。 “草根经济”的特点始终是随州经济保持稳定发展的宝贵资源和基础条件。当然，要把这些潜在的优势转化为现实竞争力，还需要我们付出艰苦的努力，保持昂扬向上的精神状态。越是在成绩面前和条件好的情况下，越是在经济发展的周期性调整中，越要把困难估计得更加充分一些，把工作思路与宏观形势联系得更加紧密一些，把应对措施制定得更加周全一些，把各项工作做得更加扎实一些，防止经济出现较大起伏和急剧回落。

2009年政府工作总的指导思想是：全面贯彻落实党的十七大、十七届三中全会和中央经济工作会议精神，以科学发展观统领经济社会发展全局，围绕“保增长、扩内需、调结构”，加快新型工业化进程，加快新农村建设，加快发展第三产业，加快推进城市建设，加快改革开放步伐，加快构建社会保障体系，力争发展速度和效益增幅高于全省平均水平，努力开创“四个随州”建设新局面，促进经济社会又好又快发展。

今年经济社会发展的主要预期目标是：地区生产总值增长11%；规模工业增加值增长16%；全社会固定资产投资增长25%以上；地方一般预算收入增长12%；外贸出口增长10%；城镇居民人均可支配收入增长8%，农民人均纯收入增长7%；社会消费品零售总额增长15%以上；居民消费价格涨幅控制在4%左右；城镇登记失业率控制在4.6%以内；单位生产总值能耗下降2.8%，二氧化硫和化学需氧量排放总量分别下降1%和2%；人口自然增长率控制在6‰以内。各项社会事业全面协调发展。

三、2009年的重点工作

2009年，政府工作的首要任务是保经济增长，重点工作是改善民生。确保实现各项发展目标，必须坚持：把加大投资与促进消费相结合，把保持增长与调整结构相结合，把扩大开放与深化改革相结合，把发展经济与改善民生相结合，坚持高标准，明确新要求，实施新举措，谋求新跨越，扎实做好七个方面工作。

（一）全力推进产业聚集，加快工业兴市步伐。继续把提升新型工业化水平放在经济发展的首要地位，尽快形成以优势产业为主导、以重点企业为支撑、以工业园区为载体的发展格局，力争规模工业增加值总量突破100亿元。一是做大做强优势产业。充分利用“中国专用汽车之都”这块金字招牌，抢抓国家扩大内需的机遇，加快低成本扩张和整合步伐，营造集团优势，突破性扩大生产规模，差异化开发新产品，培育国家级或省级名牌4个以上，力争把汽车及零部件产业，打造成随州首个年产值过百亿元的产业。积极争取与武汉东湖高新技术开发区联手，以波导、创力科等企业为载体，加快扩能改造和产品升级，加强协作配套，建设“中国光谷随州基地”；改造纺织服装工业，积极培育精品名牌，争取将电子信息和纺织服装，纳入全省重点扶持的产业集群。大力发展医药化工产业，改造提升食品加工产业，使之尽快成为工业经济的重要板块。用好用活省里关于重点产业集群的扶持政策，整合资源，延伸链条，建好“中国香菇之乡”和“中国风机城”。二是加快发展中小企业。加强政策引导，扩大中小企业融资担保机构的规模和实力，抓好中小企业成长工程，力争今年新增规模工业企业30家以上，新增纳税过500万元的企业3家。坚持质量兴市，加大名牌创建力度。突出抓好楚威公司、 星公司的上市工作，积极推进齐星公司上市前期工作，滚动培育上市后备企业，力争实现企业上市零的突破。三是掀起全民创业高潮。大力实施创业带动就业和回归创业两大工程，加大对全民创业的宣传和政策扶持力度，进一步放宽准入条件，减少准入程序，缩短准入时间，扩大优惠范围，提高优惠标准，引导上项目，鼓励办企业；探索建设 “回归工业园”，大力发展 “回归经济”。做好市内缺工企业与返乡农民工岗位对接和就业服务，通过开发公益性岗位安置困难人员就业。四是打造园区平台。在大力支持市经济开发区完善基础设施、尽快实现满园工程的同时，加快城东新区工业园的规划建设，为招商引资和新一轮的竞争发展提供平台和空间。大力支持曾都开发区、广水开发区及随州温州工业园、杨寨冶金工业园等一批特色园区建设，用硬件条件的改善来体现体制转轨后的生机与活力，形成板块聚集效应。五是高度重视企业家队伍建设。切实落实优秀企业家的政治经济生活待遇，注重培养企业管理队伍和专业技术人才队伍，教育和引导企业家进一步增强社会责任和诚信意识，不断增强搏击市场的能力。企业要度过难关，需要国家的政策支持、各级政府的关心，但更需要企业家的坚定信心和自强不息，只要迈过了当前这道坎，发展就会争取更大的主动。

（二）促进农民持续增收，加快建设社会主义新农村。把提高农民收入、夯实农业基础作为扩大内需的重要内容，巩固和发展农业农村大好形势。一是大力调整产业结构。把农业结构调整的重点放在突出特色、壮大规模、提高品质、打造品牌上，着力抓好粮食优势产业和食用菌、马铃薯、菜豌豆、优质桃、大蒜等特色产业基地建设。高度重视粮食和主要农产品供给，稳定面积、提高品质。强力推进畜牧强势产业，做好动物疫病防控工作。积极发展无公害、绿色和有机农产品，建成一批特色农业标准化种养基地，提高我市农产品市场竞争力。二是继续推进农业产业化。实施龙头带动战略，突出抓好同星农业、三友食品、神农生态、新欣农牧、广华生态等农业产业化龙头企业的提档升级。加快粮食物流园区规划建设。发展各类专业合作社，壮大经纪人队伍，提高农民在市场竞争中的组织化程度。积极争取省里支持，继续巩固200万亩菇耳林及100万亩意杨基地建设，有序扩大优质核桃、油茶等高效林业基地，提高林业效益，着力建设林业生态市。三是加强农村基础设施建设。以随南新农村实验区建设为契机，突出抓好7个乡镇、50个行政村的规划建设，打造全市农村改革发展的先行区、示范区。以“清洁工程”为重点，抓好示范区、示范镇、示范线的综合整治。以国家大幅增加对“三农”投入为契机，加快县乡道大中桥梁、通乡公路和通村水泥路建设；投资1.5亿元，继续实施千村万塘改造、高产农田建设、水库除险加固和大型灌区续建等工程，继续推进农村饮水安全工程；建成沼气池1.5万口，引导农民建设生态家园。四是认真落实各项强农惠农政策。及时启动提高粮食最低收购价的保护措施，严格抓好粮食直补、良种补贴、农机具购置补贴的发放，确保不抵扣、不拖延、不漏发。保障农民土地、林地承包和经营自主权，遵循自愿、依法、有偿的原则，积极推进土地、林地合理流转，发展多种形式的适度规模经营。实施新一轮农村危房改造工程，改善困难农户住房条件；加大扶贫力度，再启动25个贫困村整村推进工作。继续做好对口支援四川灾区重建工作。

（三）着力擦亮“两张名片”，加快发展第三产业。加大“中国优秀旅游城市”创建力度，大力提升我市在鄂西生态文化旅游圈中的竞争优势。围绕增强随州旅游的影响力、吸引力，加快编制完善旅游规划体系，强力打造“自然生态文化、历史文化”旅游品牌，加快推进“炎帝神农大洪山精品旅游线”、“大洪山历史文化旅游区”建设，把故事讲精彩，把景区建优美，把项目做丰富。加大擂鼓墩遗址保护等一批旅游项目申报力度，力争纳入全省4个旅游专项规划，为后期建设做好准备。按照4A级景区标准，加快炎帝神农故里、擂鼓墩、大洪山、新阳温泉四大景区建设；按照省级农业旅游示范点标准，加快洛阳古银杏群落、万和兰花等“五朵金花”基地建设，打造旅游农业精品亮点。加快旅游名镇、旅游名村创建步伐。引入市场机制，鼓励兴办旅游企业，进一步优化旅游发展环境。积极开拓旅游市场，将始祖、编钟、山水等优势资源整合筛编成精品项目，多渠道进行旅游形象宣传，开展“随州人游随州”、“湖北人游随州”活动，办好全球华人寻根节，逐步把“两张名片”推向全国，推向世界。继续完善城乡消费体系，多措并举拉动内需。着力培植税源型商贸企业，把政策支持与税收贡献挂钩，引入商贸大品牌。继续实施“万村千乡”和“双百”市场工程，将“家电下乡”补贴政策落实到位,推进连锁经营、物流配送向农村延伸，促进农村消费。运用现代服务理念和经营方式，改造提升商贸、餐饮等传统服务业，推动城乡集贸市场提档升级，大力发展社区商业、物业、家政等服务性消费，规范发展法律、会计、公证、资产评估等各类中介组织，促进新型消费成长。加强市场监测、信息引导，整顿和规范市场经济秩序，营造稳定、安全、放心的消费环境。加快“信用随州”建设，改善金融生态环境，确保我市成功创建A级信用市，力争当年金融机构存贷差较上年缩小50%以上。

（四）牢牢把握政策机遇，加快重点项目建设。抓经济必须抓项目，抓项目必须抓投资。今年我们将深入开展“大项目年”活动，把对上对外争取资金作为推动发展的重要动力。一是抢抓机遇做好对上争资工作。深入研读政策，找准着力点、契合点，扎实做好重大项目的包装策划和前期推进，形成“完善一批、准备一批、申报一批”的梯次接续格局。市和区市两级财政将拿出必要的资金，为争项争资前期工作提供保障，力争目前已申报的项目更多地挤进国家投资笼子，确保今年对上争取无偿投资不低于10亿元。二是抓好一批支撑投资的重大项目建设。继续实行市级领导包保责任制，落实定项目、定领导、定专班、定责任、定目标、定奖惩和一抓到底的“六定一抓”工作机制，进行经常性检查督办，保证重点项目建设的投资力度和建设进度，确保总投资38.2亿元的麻竹高速公路、总投资21.6亿元的西气东输随州段、总投资52亿元的随信铁路、总投资16.4亿元的多晶硅项目、总投资3.8亿元的风力发电以及大洪山交通设施等一批重大项目如期开工、顺利推进、加快建设。三是积极鼓励和引导社会投资。继续为社会投资创造良好环境，鼓励和引导社会投资以独资、合资、合作、联营、特许经营、项目融资等方式，参与经营性基础设施项目建设，促进固定资产投资继续保持快速增长。

（五）完善城市基础设施，加快提升管理水平。坚持“城市建设一刻不停步，城市管理一刻不放松”，不断完善城市功能，不断改善居住环境，不断提升城市品位，努力建设山水生态园林城市。一是抓基础，完善城市功能。坚持量力而行、尽力而为的原则，集中财力，统筹谋划，立足包装和整理，继续推进老城区改造，加快城区道路的提档升级，全面完成主要路段的刷黑工程。进一步改善城市布局，完成滨湖循环道改造 （包括规划建设波导大桥、白云大道二期工程、氵厥水一桥至二桥西堤建设改造），实施新火车站及配套建设，完成交通大道改造。加快便民设施建设，以滨湖体育公园建设为节点，继续加快滨湖风光带建设；以白云公园、烈士陵园改造为重点，启动城南森林公园建设；以随中灌区改造为契机，规划内河清水工程，进一步改善城市人居环境。二是抓经营，盘活城市资产。积极有效地管理土地市场，严格控制和最大效益地利用城市每一寸土地，保障国有资产增值，保障市场发展信心，促进城市建设的持续健康发展。发挥城市投资集团、交通投资公司、城市建设综合开发投资公司的主体作用，坚持项目优先、先舍后得的原则，加大市场化筹资力度，加快城市重点项目建设。加快推进环卫保洁、垃圾清运处理、园林绿化等公用事业市场化；盘活城市无形资产，多途径筹集建设资金；拓宽开发性金融合作渠道，加大与开行及其他金融机构的合作力度，力争今年城市建设投资达到10亿元以上。三是抓管理，推进“三城同创”。在新的城市总体规划的框架内，加快编制各类控制性详细规划，抓紧编制居民点规划。继续严格城市规划的刚性控制，保持对违法违规建房的高压整治态势，规范建筑市场秩序。巩固“门前四包”管理成果，切实搞好环境卫生、店面包装、车辆停靠、广告规范等综合整治；巩固城区“禁摩”成果，严防反弹。全面开展园林式单位、庭院和小区创建活动，大力实施见缝插绿、拆墙透绿工程，实现省级园林城市的创建目标。四是抓机制，促进社区发展。高度重视和改进社区工作，完善落实“权随责走、费随事转”的工作机制，在政策和经费上给予倾斜，加强社区办公设施和服务能力建设，夯实社区基层基础工作。

（六）纵深推进各项改革，加快提升开放水平。加大改革攻坚和对外开放力度，力争在重要领域和关键环节实现新的突破。一是深化改革，激发内在活力。采取更加优惠的政策，全面完成企业改革收尾工作，突出抓好重点工业企业改制后的二次招商和资产重组；以产权制度改革为重点，大力推进国有流通企业改组改制，加快粮食、交通、科技等非工业领域国有企业改革步伐；全面完成集体林权制度改革。进一步深化行政管理体制改革，严格机构编制管理，巩固和完善大部门体制。认真做好增值税转型改革，积极推进非税收入征缴改革，依法加强征管。优化收入分配结构，今年各项行政性开支和一般性开支力争保持零增长，财政增收部分主要用于保障政策性支出和奖励引导、激励发展。二是创新方式，提高招商水平。调整招商引资思路，在巩固全民招商成果的基础上，着力打造一支专业化招商队伍，采取专班招商、活动招商、对口招商、以商招商等多种形式，构建政府、企业与外商联合推动的招商模式。加快建立相对完备的专业评估体系，加强项目评估论证专家库建设，做到科学引进项目，提高专业化招商能力。舍得以资产换资本、以存量换增量，以更优惠的政策、更大的气魄招大商、引大品牌，对重大招商项目提供保姆式服务；对招商引资的工作经费给予重点保障，重奖有功之臣，力争引进内联资金43亿元以上，实际利用外资突破4000万美元。三是主动对接，承接产业转移。突出企业主体地位，以项目为纽带，全力抓好关联产业、互补产业、配套产业的承接对接，力争成批量、有针对性地引进沿海发达地区产业项目向我市扎堆转移、集群发展，力争建设几个产业转移工业园。充分发挥随州与武汉的产业互补优势，全方位、宽领域、多渠道对接武汉城市圈，力争在现代农业、汽车产业、文化旅游对接转移上取得新的进展。四是优化结构，提高出口效益。准确研判和积极应对外贸新形势，制定有效措施，优化出口结构，巩固发展机电、食用菌及兰花等特色产品出口优势。大力实施科技兴贸战略、精品名牌战略、贸易方式创新战略，加大高新技术产品出口，鼓励培育自主商标品牌、注册境外商标、建设专用车境外服务网点，积极开展电子商务和网上贸易。抢抓国家实行加工贸易差别政策机遇，加快发展加工贸易出口，努力实现对外贸易份额和效益的“双增长”。

（七）切实改善人民生活，加快建设和谐随州。把改善民生放在更加突出的位置，尽心尽力解决关系群众切身利益的实际问题。一是统筹发展各项社会事业。优先发展教育事业，深入推行素质教育，加快发展远程教育，提高农村义务教育质量；坚持开放式办学理念，继续实施普通高中错位发展战略，支持随州职业技术学院、随州技师学院、湖北现代教育集团提档升级、做大做强。加快构建公共卫生和医疗服务体系，进一步完善突发公共卫生事件应急处置机制，加大对重大传染病防控和治疗力度，努力保障食品药品质量安全和生产安全；加强医疗机构管理，切实做到安全、价廉、优质，确保市中心医院成功创建三级优秀医院；加快社区卫生服务网点建设，稳步提高农村新型合作医疗参合比例和报销标准，真正让群众看得起病、看得好病。大力发展文化体育事业，加强图书馆、乡镇文化站、健身场地等城乡文化体育设施建设，新建100家农家书屋；整顿和净化文化市场，建立网吧管理长效机制。继续实施农村电影放映工程，加快推进广播电视数字化进程，满足城乡人民多元化精神文化需求。健全利益导向政策，全力稳定低生育水平，综合治理出生人口性别比问题。积极发展妇女、儿童、老龄和残疾人事业，进一步推动人民防空建设，认真做好档案、保密、对台和民族宗教工作，发挥工会、共青团、妇联等人民团体桥梁纽带作用，调动社会各方面的积极性、创造性。二是加强就业再就业和社会保障工作。全面落实促进就业的各项政策，广泛发布就业信息，不断加大就业援助力度，动态消除“零就业家庭”，确保全市就业形势基本稳定。认真落实军转干部、复员退伍军人优抚和安置政策。加强农民工技能培训，继续组织劳务输出，新增农村劳动力转移就业1.6万人。进一步扩大社会保障覆盖面，全面实施城镇居民医疗保险，继续抓好被征地农民养老保险。不断完善最低生活保障制度，推进低保对象扩面，确保城乡特困群众基本生活。三是大力推行可持续发展战略。加强节能减排和资源节约，坚决落实项目建设环评制度，加大对重点污染企业监控力度，决不以牺牲环境为代价换取经济发展。严格保护耕地，做好高产农田和低丘岗地改造工作，促进节约集约用地。大力推进机关节能工作，促进节约型社会建设。四是全力维护社会稳定。进一步加强社会治安管理，完善突发事件应急机制，增强维护公共安全与社会稳定的能力。继续强化社会治安防控体系建设，加强农村

和社区警务工作，严厉打击黑恶

势力和“两抢一盗”等犯罪活动，

创造安宁有序的社会环境。严格

信访工作目标管理，完善领导接

访和包案处理制度，畅通群众诉

求渠道，密切党群政群关系。严格

安全生产责任制，认真开展道路水上交通、烟花爆竹、非煤矿山、建筑施工、消防等专项整治，遏制重特大事故发生，确保人民群众生命财产安全。五是切实加强精神文明建设。巩固文明城市创建成果，广泛开展“社会责任感”教育，全面开展 “十佳文明诚信企业”、“十佳文明出租车”等一批“十佳”先进典型评选活动。以文明村镇创建为重点，潜移默化提高农民文明素质；以社会主义核心价值体系为重点，加强未成年人理想信念教育和思想道德建设。进一步做好国防动员和民兵预备役工作，深入开展“双拥”共建，巩固和发展军政军民团结。

四、全面加强政府自身建设

各级政府肩负着时代的重托、发展的使命，须臾不可懈怠，丝毫不敢马虎。只有全面加强自身建设，不断提高执政能力，切实做到日常性工作求规范、重点性工作求创新、全局性工作争一流，才能不辜负全市人民的期望和重托。

（一）坚持改善民生办实事。实现好、维护好、发展好人民群众的根本利益，是政府一切工作的出发点和落脚点。市政府将坚持以人为本，克服资金困难，努力为市民办好以下10件实事：（1）全面实施创业带动就业工程，对回乡创业实行注册登记零收费，实现再就业5000人，带动城镇新增就业1.5万人；（2）基本实现城镇医疗保险全覆盖，总数达到60万人以上；（3）改善农村寄宿制学校生活条件，提高对寄宿制学校生活困难学生的救助标准；（4）完成白云公园改造，并免费向市民开放；（5）解决农村16万人口的饮水安全问题；（6）完成150个以上建制村村庄整治；（7）建成500套廉租房，加快经济适用房、经济租赁房建设；（8）加快农村医疗卫生机构建设，改造5个乡镇卫生院、50个村级医疗点；（9）兴建市社会福利院；（10）建成市中医院住院大楼。

（二）坚持强化责任促执行。继续强力推进政府效能和作风建设，建立健全“四种机制”，提高政府的公信力、执行力，提高政府服务人民群众、服务市场主体的能力。一是建立健全民主决策机制。进一步完善重大事项集体决策制度、专家咨询制度、社会公示和听证制度、决策责任制度，决策之前讲民主，决策之后抓落实，保证政府各项工作集思广益、科学推进。二是建立健全层级负责机制。各级政府及政府部门负责同志要带头深入一线，靠前指挥，多到条件差、困难多的地方开展工作，帮助解决问题，以实实在在的作风，促进实实在在的发展；全体政府工作人员都应增强责任感、使命感，立足本职，调整状态，敢于负责、敢于突破，形成层层负责、层层抓落实的工作格局。三是建立健全畅通运作机制。以优化政务环境为重点，大力推广“马上就办、有请必复”的办事理念和机关文化，定下来的事雷厉风行，看准了的事一抓到底。建立首问负责制、限时办结制、督查通报制和大企业服务直通车制度，完善运作体系，加快行政提速，切实做到重心下移、服务前移、提高效率。大力精简会议和文件，大力精简审批环节，再造审批流程，力争把各类行政性收费降至最低、对重点工程建设直至实现本级行政规费零收费。四是建立健全问责追究机制。把加强作风建设，从整纲肃纪向行政问责延伸，形成有权必有责、有责必施问、有错必追究的工作机制，政府将对本年度的各项重点工作逐一分解，抓好跟踪督查，定期通报，确保落实。

（三）坚持解放思想求创新。以深入开展科学发展观学习实践活动为契机，加强政策理论、市场经济和相关专业知识的学习，把解放思想的成果落实到解决具体问题上，善于审时度势，以创新的思路去破解经济结构不优、建设资金不足、农民增收渠道不宽等难题，多做打基础、管长远的工作。大力倡导逆向思维、危中寻机、困中求进的精神，关键时刻能够扛得起、顶得住、上得去；大力倡导敢为人先、争创一流的精神，鼓励冒尖，激励探索，宽容失败，努力做到发展有新思路、工作有新成效。

（四）坚持依法行政抓规范。加快推进法治政府建设，严格依照法定权限和程序行使权力、履行职责、接受监督，切实规范各级政府及其工作人员的行为。认真执行人大及其常委会的决议、决定，主动加强与人民政协的协商，扎实办好建议、提案。依法开展行政复议、行政应诉工作，严厉查处行政执法环节的不作为、乱作为，保障公民合法权益。进一步发展基层民主，健全完善村民自治、居民自治、职工代表大会等制度，不断推进基层政权建设。

（五）坚持廉洁从政树形象。严格落实党风廉政建设责任制，坚持教育、制度、监督并举，做到关口前移、防范在先，重点防治工程招投标、土地出让、产权交易、政府采购等领域的不正之风，不断完善惩治和预防腐败体系。加大审计监督、行政监察力度，加大腐败案件查处力度，深入开展民主评议政风行风活动，严肃查处损害群众利益的违纪违法行为。大力提倡艰苦创业、厉行节约和勤俭办一切事情，树立人民政府的良好形象。

各位代表，面对新的形势和任务，我们倍感使命光荣、责任重大，倍感机遇难得、催人奋进。让我们在市委的坚强领导下，依靠全市人民，凝聚各方力量，坚定信心，抢抓机遇，继往开来，务实进取，谱写人民美好生活新篇章，以优异的成绩向建国60周年献礼！
